# Diet, Secondhand Smoke, and Glycated Hemoglobin (HbA1c) Levels among Singapore Chinese Adults

**DOI:** 10.3390/ijerph16245148

**Published:** 2019-12-17

**Authors:** Brianna F. Moore, Lesley M. Butler, Annette M. Bachand, Agus Salim, Stephen J. Reynolds, Renwei Wang, Tracy L. Nelson, Jennifer L. Peel, Sharon E. Murphy, Woon-Puay Koh, Jian-Min Yuan, Maggie L. Clark

**Affiliations:** 1 Department of Epidemiology, Colorado School of Public Health, Aurora, CO 80045, USA; 2 The UPMC Hillman Cancer Center, University of Pittsburgh, Pittsburgh, PA 15232, USA; butlerl3@upmc.edu (L.M.B.); wangr2@upmc.edu (R.W.); yuanj@upmc.edu (J.-M.Y.); 3 Department of Epidemiology, Graduate School of Public Health, University of Pittsburgh, Pittsburgh, PA 15261, USA; 4 Ramboll U.S. Corporation, 28 Amity Street Suite 2A, Amherst, MA 01002, USA; Annette.Bachand@colostate.edu; 5 Department of Environmental and Radiological Health Sciences, Colorado State University, Fort Collins, CO 80523, USA; stephen.reynolds@colostate.edu (S.J.R.); Jennifer.Peel@ColoState.EDU (J.L.P.); Maggie.Clark@ColoState.EDU (M.L.C.); 6 Department of Mathematics and Statistics, La Trobe University, Bundoora, Victoria 3086, Australia; A.Salim@latrobe.edu.au; 7 Baker Institute for Heart and Diabetes, Melbourne, Victoria 3004, Australia; 8 Department of Health and Exercise Science, Colorado State University, Fort Collins, CO 80523, USA; Tracy.Nelson@colostate.edu; 9 Masonic Cancer Center, University of Minnesota, Minneapolis, MN 55455, USA; murph062@umn.edu; 10 Department of Biochemistry, Molecular Biology and BioPhysics, University of Minnesota, Minneapolis, MN 55455, USA; 11 Health Services and Systems Research, Duke-NUS Medical School, Singapore 169857, Singapore; woonpuay.koh@duke-nus.edu.sg; 12 Saw Swee School of Public Health, National University of Singapore, Singapore 117549, Singapore

**Keywords:** diet, antioxidants, omega-3 polyunsaturated fatty acids, cotinine, HbA1c, interaction

## Abstract

The combination of poor diet and exposure to secondhand smoke may increase hemoglobin A1c (HbA1c) levels, but few studies have explored this interaction. We explored an interaction among 574 never-smoking adults from the Singapore Chinese Health Study. At baseline (age 59 ± 8 years), intakes of omega-3 polyunsaturated fatty acids, vitamin C, vitamin E and fiber were estimated using a modified food frequency questionnaire. At follow-up (age 64 ± 9 years), HbA1c and cotinine were measured. A product term between cotinine (above or below the median value) and each nutrient (high or low intake) was included in separate linear regression models with HbA1c as the outcome. HbA1c among those with high cotinine and low omega-3 polyunsaturated fatty acids intakes were higher than would be expected due to the individual effects alone (*p*-for-interaction = 0.05). Among those with lower intakes of omega-3 polyunsaturated fatty acids, high cotinine levels were associated with 0.54% higher HbA1c levels (95% confidence interval [CI]: 0.02, 1.06). Conversely, among those with higher intakes of omega-3 polyunsaturated fatty acids, HbA1c differ not differ by exposure (−0.09%; 95% CI: −0.45, 0.30). No evidence of interaction was observed for other nutrients. Diets high in omega-3 polyunsaturated fatty acids may ameliorate secondhand smoke-induced increases in HbA1c.

## 1. Introduction

Globally, one in three children and non-smoking adults are involuntarily exposed to secondhand smoke on a daily basis [1]. The high occurrence of this exposure is worrisome because exposure to secondhand smoke may increase the risk for coronary heart disease by 30% [2], and type 2 diabetes by 21% [3]. Although these associations have been widely studied, the impact of exposure to secondhand smoke on early indicators of chronic disease has not been thoroughly explored.

Hemoglobin A1C (HbA1c) is an important early indicator of cardiovascular disease and type 2 diabetes [4,5]. Two previous studies have reported positive associations between higher levels of cotinine (the major metabolite of nicotine) [6] with increased HbA1c levels [7,8]. Both of these studies have been conducted among United States adults who participated in the National Health and Nutrition Examination Survey (NHANES). The extent to which this exposure is associated with HbA1c levels among adults in other countries is unknown.

The mechanisms linking exposure to secondhand smoke with increased HbA1c levels are also poorly understood. One potential mechanism is oxidative stress. Secondhand smoke induces oxidative stress, which has been implicated in the glycation process [9]. Certain dietary nutrients, such as omega-3 polyunsaturated fatty acids, antioxidants, or dietary fiber, may limit secondhand smoke-induced oxidative stress responses [10]. Previous studies have reported that high intakes of these nutrients may ameliorate the effects of exposure to secondhand smoke on HbA1c levels among children [11] and on cardiovascular disease among adults [12,13]. These findings highlight the need to examine whether diet may limit the effect of exposure to secondhand smoke on HbA1c levels among adults.

This analysis was conducted among a subset of never smoking adults enrolled in a prospective cohort: the Singapore Chinese Health Study. The objective of our study was to explore the potential interaction between diet and exposure to secondhand smoke on HbA1c levels among Singapore Chinese adults. 

## 2. Methods

### 2.1. Study Population

The Singapore Chinese Health Study is a large, population-based prospective cohort study [14]. Participants were recruited between 1993 and 1998. Participants were eligible if they were between the ages of 45–74 at the time of enrolment, were permanent residents or citizens of Singapore, and resided in government-built housing. Demographic and dietary information were collected from each participant at the baseline interview. Between 1994 and 2005, blood and urine samples were collected from a subsample of the participants. During the first follow-up interview between 1999 and 2004, participants were asked to self-report their exposure to secondhand smoke. Participants are followed through time via the Singapore Registry of Births and Deaths, the Hospital Discharge Database and Singapore Cancer Registry. These databases are then linked to the original cohort data. Attrition in the Singapore Chinese Health Study is extremely low. As of December 2014, only 47 (0.7%) participants were lost to follow-up due to migration out of Singapore.

The study protocol was reviewed and approved by the Institutional Review Boards of the National University of Singapore and the University of Pittsburgh. Written informed consent was obtained from all participants prior to data collection.

### 2.2. Inclusion Criteria

**Figure 1:**
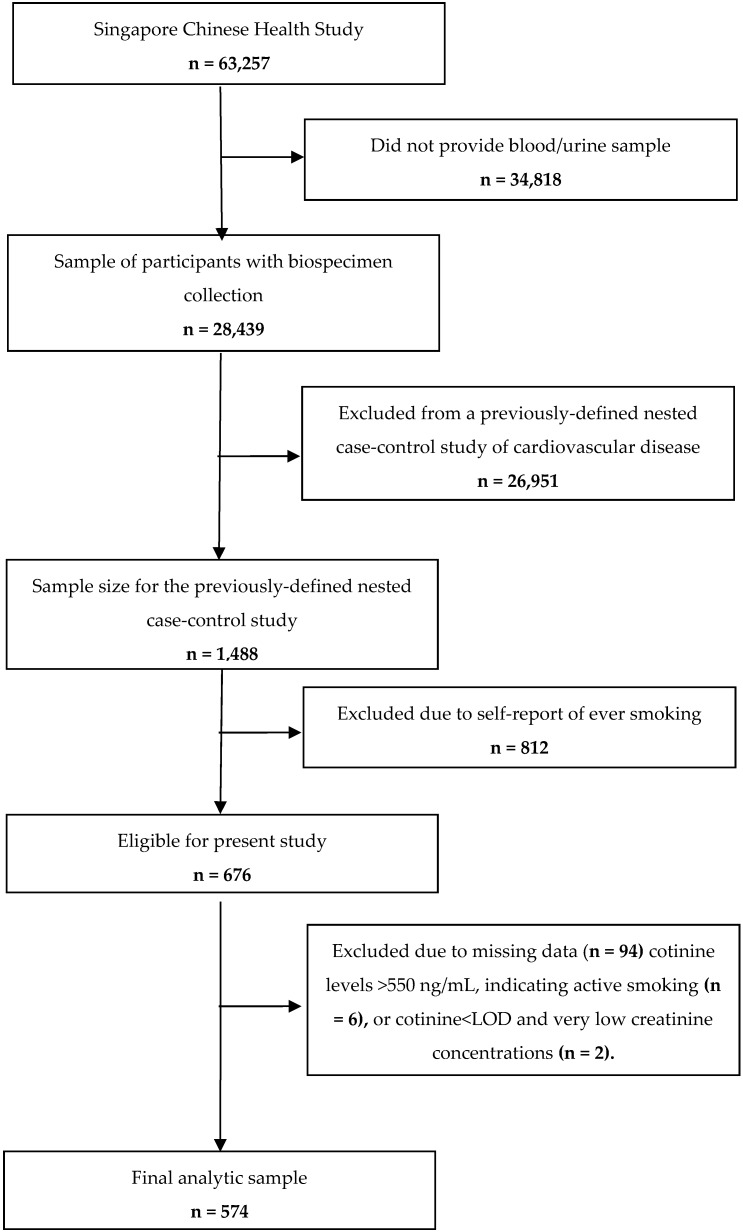
Study population and exclusions.

The Singapore Chinese Health Study recruited 63,257 participants from 1993 to 1998 (Figure 1). Our study was conducted using data from a previously defined, nested case-control study of cardiovascular disease, as described by Sun et al. [15]. These cases and controls were selected from participants who provided blood, and did not have a history of physician-diagnosed cardiovascular disease or stroke (either self-reported or ascertained through linkage with the Hospital Discharge Database). The eligible cases for the present study were incident acute myocardial infarction that occurred during follow-up via linkage with the three databases (described above). All eligible cases were enrolled. Using a risk-set sampling approach [16], 744 controls were selected for each of the 744 cases of fatal coronary heart disease or non-fatal myocardial infarction. 

Controls were participants who were alive and free of coronary heart disease at the date of the diagnosis or date of death among cases. Controls were matched on sex, dialect group (Hokkien, Cantonese), date of birth (±5 years), date of recruitment (±2.5 years), and date of blood collection (±6 months).

A subset of the never-smoking case-control pairs were selected to evaluate the associations between secondhand smoke and incident coronary heart disease and biomarkers of chronic disease risk (e.g., HbA1c). A total of 277 existing. case-control. never smoking pairs were identified. To increase our sample size, we included an additional 52 case-control pairs who were matched on the criteria as described above. and 9 never smoking cases-control pairs who were matched on sex, dialect group and year of birth, but not matched on year of recruitment and date of biospecimen collection. Urinary biospecimen samples from this dataset were analyzed for cotinine. Of the available 676 participants (338 cases and 338 controls), 582 participants (329 cases and 253 controls) had complete data on the exposures, outcomes and covariates. We further excluded six participants with evidence of active smoking, defined as having a urinary cotinine concentration >31.5 ng/mL [17] and two participants that had cotinine below the laboratory limit of detection, but that were categorized in the high cotinine group after creatinine adjustment (see approach below). Therefore, the final analytic sample for this cross-sectional analysis was 574 participants (324 cases and 250 controls).

### 2.3. Exposure to Secondhand Smoke

Total cotinine was measured in spot urine samples using liquid chromatography linked to tandem mass spectrometry. The limit of detection for cotinine was 0.20 ng/mL. To account for variation in dilution of the spot urine samples, urinary cotinine was corrected by urinary creatinine [18]. A limited number of participants had cotinine levels below the limit of detection (n = 40, 7%). Cotinine levels were heavily skewed right, even after non-linear transformation, which led to a violation of the assumption of normality for the residuals in linear regression analyses. Currently, there are no established cut-offs for cotinine to differentiate low or high exposure to secondhand smoke. Therefore, we dichotomized cotinine as low exposure and high exposure based on a median-split. This data-driven approach was employed to increase statistical power, and has been used in a previous study evaluating a similar research question [19]. The cotinine categories were defined as follows: low exposure (cotinine < 0.01 ng/mg creatinine [the median value], including those below the limit of detection) and high exposure (cotinine ≥ 0.01 ng/mg creatinine). 

Self-report of exposure to secondhand smoke was ascertained at the first follow-up visit. Participants were asked to self-report whether they lived with a smoker who smoked in the home on a daily basis (yes or no). 

### 2.4. HbA1c Assessment

HbA1c was measured on red blood cells isolated from whole blood samples of participants in our analytic sample. HbA1c measurements were performed on the Bio-Rad Variant II Analyzer (Hemel Hempstead, United Kingdom) and were quantified using high-performance liquid chromatography. 

### 2.5. Dietary Assessment

A 165-item modified quantitative food frequency questionnaire was administered at the time of enrolment [20]. Nutrient values were primarily obtained from the United States Department of Agriculture National Nutrient Database for Standard Reference [21], as described previously [20]. We evaluated diet in terms of individual nutrients that may alleviate secondhand smoke-induced oxidative stress [10]. Dietary fiber, vitamin C, vitamin E and omega-3 polyunsaturated fatty acids were dichotomized using categories, as previously described within this population [12]: low (the lowest quartile of intake) and high (the second through fourth quartiles of intake).

### 2.6. Covariates

At the baseline interview, information about the participant’s age, sex, dialect and education was collected. Self-reported heights and weights were collected through baseline questionnaires. Although older adults tend to overestimate height and underestimate weight, previous studies have reported a high level of agreement between measured and reported height and weight data (correlation coefficients ranging from 0.85 to 0.96) [22,23]. Of the 63,257 participants from the original cohort study, 9781 participants (15.5%) were missing weight data, 97 participants (0.2%) were missing height data, and 192 participants (0.3%) were missing both weight and height data. Weights and/or heights were imputed for these participants using linear regression methods described elsewhere [24]. Body mass index (BMI) was calculated by dividing weight (kg) by height (m) squared. Previous work in this cohort has demonstrated that the results were nearly identical after including and excluding participants with imputed BMI values [24]. Overweight and obesity was determined using cut-off points for BMI that are specific to Asian populations, as recommended by the World Health Organization (WHO) Expert Consultation [25]. The BMI categories were as follows: lean (BMI < 23 kg/m^2^), overweight (BMI ≥ 23 kg/m^2^ and <27.5 kg/m^2^) and obese (BMI ≥ 27.5 kg/m^2^). These categories correspond to an elevated risk for chronic disease among Asian populations [26].

### 2.7. Statistical Analysis

This analysis was conducted among a sample from a previously defined, nested, case-control study with a different health endpoint (coronary heart disease). Ignoring the matched case-control study design could lead to biased effect estimates, because coronary heart disease may be associated with our exposure and outcome of interest [27]. Therefore, our analyses incorporated sampling weights that were designed to account for the probability of a control being selected to participate in the previously defined nested case-control study [28,29]. This approach has been implemented in a published study that analyzed case-control data for additional outcomes [30]. 

Independent samples t-tests were used to examine differences in means, and chi-square tests were used to examine differences in proportions of the covariates across the cotinine categories. The correlations between individual nutrients were calculated using the Spearman’s rank correlation coefficient.

Linear regression models examined the main effect association between cotinine categories (low or high) and HbA1c levels. Our minimally-adjusted model included sex, date of birth, date of recruitment, date of biospecimen collection and dialect (Cantonese, Hokkien). Our fully-adjusted model additionally included education (no formal education, primary education, secondary education) and BMI (kg/m^2^) at the follow-up visit in the regression model. We examined the interaction between cotinine categories (low or high) and each of the selected nutrients (high or low intake) on HbA1c levels by including an additional product term in separate models. Our final models present adjusted means, beta coefficients and corresponding 95% confidence intervals (CIs).

All analyses were performed using Stata version 13 (Stata-Corp LP, College Station, TX, USA). An alpha level of <0.05 was used to determine statistical significance. 

### 2.8. Secondary Analyses

The main effects and interaction analyses were repeated with a self-report of household smokers as the exposure of interest. Additionally, we adjusted for total caloric intake to assess the impact of confounding by diet. Finally, we explored an interaction between cotinine and a vegetable–fruit–soy dietary pattern, which was previously identified through a principal components analysis (PCA) [31]. Our rationale for considering these analyses as ‘secondary analyses’ was due to our a priori hypothesis that specific nutrients, rather than overall diet, may protect against exposure to secondhand smoke. 

## 3. Results

Table 1 presents means and proportions for HbA1c, dietary nutrients and covariates for all participants, and for participants with low or high urinary cotinine levels. The mean HbA1c level was 6.47%. Approximately 17% of the participants reported living with any household smokers. Women represented 61% of the overall study population. Approximately 23% of the participants had received no formal education. The mean age at baseline was 59 years (standard deviation [SD]: 8 years). The mean BMI at follow-up was 23 kg/m^2^. Approximately 39% of the participants were classified as overweight and an additional 18% were classified as obese at follow-up. 

Adults with high cotinine levels tended to self-report living with a household smoker more often than adults with low cotinine levels (*p* < 0.01). Adults with low and high cotinine levels were similar with respect to HbA1c levels (*p* = 0.33), sex (*p* = 0.08), dialect (*p* = 0.39) and BMI levels at baseline (*p* = 0.16) and follow-up (*p* = 0.33). There were differences in the highest education level obtained across cotinine categories (*p* = 0.03). Adults with high cotinine levels were younger than adults with low cotinine levels at baseline (*p* < 0.01), the time of follow-up (*p* < 0.01), and the biospecimen collection (*p* < 0.01). With respect to diet, adults with low and high cotinine levels had similar dietary intakes of omega-3 polyunsaturated fatty acids (*p* = 0.69), Vitamin C (*p* = 0.62), Vitamin E (*p* = 0.88) and fiber (*p* = 0.51). 

### 3.1. Exposure to Secondhand Smoke

The mean creatinine-adjusted cotinine levels were significantly higher among those living with a household smoker (0.03 ng/mg creatinine, SD: 0.03) as compared to those who reported no household smokers (0.01 ng/mg creatinine; SD: 0.03; *p* < 0.01). 

### 3.2. Diet

The individual nutrients were correlated. Dietary fiber was strongly correlated with omega-3 polyunsaturated fatty acids, vitamin C and vitamin E (Spearman’s rank correlation coefficients of 0.66, 0.73, and 0.77, respectively). 

Vitamin C was moderately correlated with omega-3 polyunsaturated fatty acids and vitamin E (Spearman’s rank correlation coefficients of 0.47 and 0.63, respectively). Vitamin E and omega-3 polyunsaturated fatty acids were strongly correlated (Spearman’s rank correlation coefficient: 0.77).

### 3.3. Main Effects

Cotinine levels were not independently associated with HbA1c (Table 2). There was virtually no difference in the adjusted mean HbA1c level across the cotinine categories (adjusted beta coefficient: 0.06%; 95% CI: −0.26, 0.37). Therefore, we conducted interaction analyses within the minimally-adjusted model.

### 3.4. Interaction Analyses

In the minimally-adjusted model, HbA1c levels among those with high cotinine levels and low intakes of omega-3 polyunsaturated fatty acids were higher than would be expected due to the individual effects alone (Table 2; *p* for interaction = 0.05). Among those with low intakes of omega-3 polyunsaturated fatty acids, the adjusted mean HbA1c level was 0.54% higher among those with high as compared to low cotinine levels (95% CI: 0.02, 1.06). Among those with high intakes of omega-3 polyunsaturated fatty acids, there was no difference in the adjusted mean HbA1c level across the cotinine categories (adjusted beta coefficient: −0.09%; 95% CI: −0.45, 0.30). A similar pattern was also observed for the interaction between the cotinine and vitamin C, but the interaction was not statistically significant. Dietary intakes of fiber or vitamin E did not appear to modify the association between cotinine levels and HbA1c. 

### 3.5. Secondary Analyses

Consistent with the cotinine analyses, living with a household smoker was not independently associated with HbA1c levels (Table 3). There was no evidence that dietary intakes of omega-3 polyunsaturated fatty acids, vitamin C, fiber, or vitamin E modified the association between any self-report of household smokers and HbA1c levels (Table 3). 

When we included total caloric intake (kCal/day) in the models, the interaction results were consistent with the null (results not presented). The results for the interaction between cotinine categories and the vegetable–fruit–soy pattern were also consistent with the null (results not presented).

## 4. Discussion

The association between high (as compared to low) cotinine and HbA1c may be limited to those with low intakes of omega-3 polyunsaturated fatty acids. It is not clear why the adjustment for total caloric intake in secondary analyses did not support the results of our primary interaction model. Although further research should evaluate this question, our primary approach may align more closely with studies evaluating the protective role of dietary supplements (regardless of total energy intake) on the detrimental health impacts of exposure to various sources of air pollution [10]. Our results do not support the hypothesis that other nutrients, nor the vegetable–fruit–soy pattern, modified the association between cotinine and HbA1c. 

Our interaction results are supported by previous work. We previously reported that high intakes of DHA and EPA may protect against the adverse metabolic responses triggered by exposure to secondhand smoke among children [11,32,33]. Additionally, two prospective cohort studies among non-smoking adults reported that the omega-3 polyunsaturated fatty acids found in fish modified the positive association between smoking and coronary heart disease incidence [34,35]. 

Our findings point toward oxidative stress as an important biological mechanism. Secondhand smoke is an abundant source of reactive oxygen species (ROS) and contributes to oxidative stress [36]. Remarkably, secondhand smoke may increase the formation of isoprostane 8-epi-PGF_2a_, a biomarker of oxidative injury, by at least 30% after only 60 minutes of exposure [37]. The oxidative stress response induced by exposure to secondhand smoke may be partially blocked by diet. High intakes of omega-3 polyunsaturated fatty acids may counteract the oxidative stress response by improving the body’s antioxidant–prooxidant balance [10], which is supported by both animal and human studies [38,39].

Currently, there are no dietary recommendations for total omega-3 polyunsaturated fatty acids. However, there are recommendations for three specific omega-3 polyunsaturated fatty acids: alpha-linolenic acid (ALA), eicosapentaenoic acid (EPA) and docosahexaenoic acid (DHA). The Food and Nutrition Board of the Institute of Medicine (now called the National Academy of Medicine) recommends that men consume 1.6 g of ALA per day, and women consume 1.1 g of ALA per day [40]. The 2015–2020 Dietary Guidelines for Americans recommend that adults consume eight ounces per week of a variety of seafood, in order to provide approximately 0.25 g per day of EPA and DHA [41]. In this analysis, exposure to secondhand smoke was associated with an increase in HbA1c levels only among those with low (or inadequate) intakes of total omega-3 polyunsaturated fatty acids (0.18–0.59 g/day). Conversely, among those with high intakes of total omega-3 polyunsaturated fatty acids (0.60–2.94 g/day), there was no difference in HbA1c levels between exposed and non-exposed adults. These interaction results suggest that adequate intakes of omega-3 polyunsaturated fatty acids may reduce the adverse effects of secondhand smoke exposure on HbA1c levels.

Previous studies among adults in the United States have reported a positive association between cotinine and HbA1c levels [7,8], whereas our findings were consistent with the null. Several factors could explain this discrepancy are: First, the previous studies were conducted using a large sample of adults who participated in NHANES. Clair and colleagues [7] detected a mean difference of 0.02% in HbA1c levels between 7350 adults with low serum cotinine levels and 4946 adults with high serum cotinine levels. Similarly, Kermah and colleagues [8] detected a mean difference of 0.03% in HbA1c levels between 2835 adults with low serum cotinine levels and 1759 adults with high serum cotinine levels. In the present study, we observed a mean difference of similar magnitude (mean difference of 0.06%) between 309 adults with low cotinine levels and 265 adults with high cotinine levels. However, the effect estimates were not statistically significant. Second, our interaction results suggest that the association between exposure to secondhand smoke, and HbA1c may depend upon diet. We might expect the effect estimates to vary across studies, since there are known regional differences in dietary intakes, but the previous studies did not stratify by nutrient intakes.

Our study has a number of strengths and some limitations. The use of the previously defined nested case-control study is an important strength. The nested case-control was designed to leverage the ongoing, well-characterized Singapore Chinese Health Study, and combine it with thorough assessments of novel biomarkers. Our use of the nested case-control design minimized selection bias and improved our ability to establish temporality. Furthermore, the controls were selected to be representative of the source population (e.g., never smoking adults free of acute myocardial infarction), increasing the generalizability of our results. However, the sample size selection of 744 incident cases of acute myocardial infarction and 744 matched controls, which was determined based on previous power calculations and available funding, may have limited our ability to detect smaller effect sizes.

Our exposure assessment was limited to a one-time measurement of cotinine. Due to the relatively short half-life (~18 h), cotinine may fail to accurately capture exposure to secondhand smoke when the exposure is intermittent (e.g., only on the weekends) [17]. It is not clear whether a dose-response relationship exists between cotinine and HbA1c. Clair et al. [7] reported a dose-response relationship, whereas Kermah et al. [8] and Moore et al. [11] did not. In this analysis, we were unable to explore the potential dose-response relationship because the continuous cotinine variable was heavily skewed, even after transformation, and because we lacked the statistical power to explore more than two categories of cotinine. Since there are no established cut-offs to differentiate high and low exposure, our data-driven approach may not have captured biologically meaningful differences in exposure. Furthermore, our cotinine and self-report results did not agree. This discrepancy could be explained by exposure misclassification, since self-report of household smokers may have failed to accurately capture exposure from other sources. Such potential misclassification would be non-differential with respect to the outcome, and therefore, may have biased associations towards the null.

Another potential limitation of our approach is the dietary assessment. The adults in our study may have failed to accurately report food frequency or quantity, particularly if foods are not explicitly listed on the food frequency questionnaires [42]. To address this limitation, the Singapore Chinese Health Study validated this questionnaire within this population [20]. This approach is considered to be a reliable method for evaluating usual dietary intake in large observational studies [43]. However, there remains a concern about the validity of food frequency questionnaires for estimating nutrient intakes. In an ethnically diverse population of adults in Singapore, Whitton and colleagues [1] conducted a study to compare nutrient intakes derived from a 163-item modified FFQ with that of a 24-h dietary recall and various biomarkers. The strongest agreement occurred when comparing the food frequency questionnaire to the 24-h dietary recall. Moderate correlations were observed for fiber (r = 0.49–0.56) and vitamin C (r = 0.43–0.50). When comparing the food frequency questionnaire to related biomarkers, low correlations were observed for the association between total polyunsaturated fat and plasma EPA + DHA (r = 0.12–0.15). However, it should be noted that the correlation between omega-3 polyunsaturated fatty acids and plasma EPA + DHA was not examined. In light of these limitations, we acknowledge that our reliance on the food frequency questionnaire to generate nutrient data may have led to measurement error.

## 5. Conclusions

In the present analysis, we demonstrated that the positive association between cotinine and HbA1c may be limited to those with inadequate intakes of omega-3 polyunsaturated fatty acids. One potential strategy for preventing population-level increases in HbA1c would be to target both exposure to secondhand smoke and diet in the same public health campaign. However, since avoiding exposure to secondhand smoke and increasing omega-3 polyunsaturated fatty acid intake may not be feasible for all populations, public health campaigns should give special consideration to populations with more than one risk factor for metabolic disorders.

## Figures and Tables

**Table 1:** Characteristics of study participants according to cotinine categories.

Outcome, Exposure Variables, and Covariates		Cotinine Categories ^a^		
	All (n = 574)	Low (n = 309)	High (n = 265)	*p*-Value
**HbA1c, %**	6.47 ± 1.52	6.51 ± 1.45	6.42 ± 1.60	0.33
**Cotinine, ng/mg creatinine**	0.01 ± 0.02	0.005 ± 0.0001	0.025 ± 0.001	<0.01
**Currently living with a household smoker**				
No	83%	91%	75%	<0.01
Yes	17%	9%	25%	
**Covariates**				
Male	39%	38%	44%	0.08
Dialect				
Cantonese	51%	50%	53%	0.39
Hokkien	49%	50%	47%	
Education				
No formal education	23%	28%	17%	0.03
Primary	43%	37%	49%	
Secondary	35%	36%	33%	
Age at baseline, years	59 ± 8	60 ± 8	58 ± 8	<0.01
Age at follow-up, years	65 ± 8	66 ± 8	64 ± 8	<0.01
Age at biospecimen collection, years	64 ± 9	65 ± 8	63 ± 9	<0.01
BMI at baseline, kg/m^2^	23 ± 4	24 ± 4	23 ± 3	0.16
^b^ BMI categories at baseline				
Lean (BMI < 23 kg/m^2^)	44%	43%	44%	0.19
Overweight (BMI ≥ 23 kg/m^2^ and <27.5 kg/m^2^)	44%	42%	46%	
Obese (BMI ≥ 27.5 kg/m^2^)	12%	15%	10%	
BMI at follow-up, kg/m^2^	23 ± 4	24 ± 4	23 ± 4	0.33
^b^ BMI categories at follow-up				
Lean (BMI < 23 kg/m^2^)	43%	43%	43%	0.84
Overweight (BMI ≥23 kg/m^2^ and <27.5 kg/m^2^)	39%	28%	40%	
Obese (BMI ≥ 27.5 kg/m^2^)	18%	18%	17%	
**Dietary nutrients**				
Omega-3 polyunsaturated fatty acids, g/day	0.88 ± 0.41	0.87 ± 0.43	0.88 ± 0.37	0.69
Vitamin C, mg/day	106 ± 128	106 ± 128	108 ± 130	0.62
Vitamin E, mg/day	12.7 ± 41.9	11.6 ± 37.8	13.9 ± 45.2	0.88
Fiber, g/day	13.0 ± 5.8	13.1 ± 6.2	12.9 ± 5.5	0.51

Abbreviations: BMI, body mass index; HbA1c, glycated hemoglobin. Continuous variables are expressed as means ± standard deviation (SD). Categorical variables are express as proportions of column totals. Independent samples t-tests were used to examine differences in means and chi-square tests were used to examine differences in proportions across the cotinine categories. ^a^ Cotinine categories were low (urinary cotinine < 0.01 ng/mg creatinine) and high (urinary cotinine ≥ 0.01 ng/mg creatinine). ^b^ Overweight and obesity was determined using cut-off points for BMI that are specific to Asian populations.

**Table 2:** Adjusted ^a^ means HbA1c according to cotinine ^b^ and nutrient categories ^c^.

Dietary Nutrients	Cotinine	n	Adjusted Mean (95% CI)	Adjusted Coefficient within Nutrient Strata (95% CI)
None (Main effect) ^d^	Low	309	6.11 (6.05, 6.17)	N/A
	High	265	+0.06 (−0.26, 0.38); *p* = 0.70	
**Omega-3 Fatty Acids**				
High (0.60–2.94 g/day)	Low	228	6.17 (5.91, 6.42)	Reference
	High	200	6.07 (5.81, 6.32)	−0.09 (−0.45, 0.30); *p* = 0.67
Low (0.18–0.59 g/day)	Low	81	5.90 (5.71, 6.08)	Reference
	High	65	6.44 (5.91, 6.99)	0.54 (0.02, 1.06); *p* = 0.04
*p* for interaction: 0.05 ^e^				
**Vitamin C**				
High (44.60–1250.35 mg/day)	Low	238	6.11 (5.86, 6.37)	Reference
	High	200	6.16 (5.92, 6.40)	0.04 (−0.31, 0.38); *p* = 0.83
Low (5.58–44.59 mg/day)	Low	71	6.10 (5.63, 6.56)	Reference
	High	65	6.32 (5.85, 6.79)	0.42 (−0.25, 1.09); *p* = 0.22
*p* for interaction: 0.47				
**Vitamin E**				
High (4.07–415.69 mg/day)	Low	224	6.06 (5.83, 6.31)	Reference
	High	201	6.16 (5.88, 6.44)	0.10 (−0.23, 0.50); *p* = 0.47
Low (0.80–4.06 mg/day)	Low	85	6.24 (5.82, 6.66)	Reference
	High	64	6.22 (5.75, 6.68)	−0.03 (−0.63, 0.57); *p* = 0.92
*p* for interaction: 0.75				
**Fiber**				
High (8.70–42.33 g/day)	Low	231	6.19 (5.95, 6.42)	Reference
	High	195	6.18 (5.88, 6.48)	0.02 (−0.37, 0.40); *p* = 0.93
Low (2.58–8.69 g/day)	Low	78	5.86 (5.46, 6.26)	Reference
	High	70	6.13 (5.78, 6.48)	0.28 (−0.26, 0.82); *p* = 0.19
*p* for interaction: 0.40				

Abbreviations: CI, confidence interval; HbA1c, glycated hemoglobin. ^a^ The linear regression models adjusted for sex, dialect, date of birth, date of recruitment and date of biospecimen collection. ^b^ Cotinine categories were low (urinary cotinine < 0.01 ng/mg creatinine) and high (urinary cotinine ≥ 0.01 ng/mg creatinine). ^c^ Nutrient categories were low (lowest quartile) and high (second through fourth quartiles). ^d^ In addition to the minimal set of covariates, the fully adjusted model included education and BMI at follow-up in the model. ^e^ The *p*-values for interaction were generated by adding a product term between the cotinine and nutrient categories into separate models.

**Table 3:** Adjusted ^a^ means and mean differences in HbA1c according to self-report of household smokers and nutrient categories ^b^.

Dietary Nutrients	Household Smokers	n	Adjusted Means and Differences (95% CI)	Adjusted Coefficient within Nutrient Strata (95% CI)
None (Main effect) ^c^	None	479	6.08 (5.89, 6.21)	N/A
	Any	95	+0.12 (−0.32, 0.56); *p* = 0.61	
**Omega-3 Fatty Acids**				
High (0.60–2.94 g/day)	None	358	6.07 (5.86, 6.26)	Reference
	Any	70	6.33 (5.85, 6.83)	0.26 (−0.24, 0.88): *p* = 0.37
Low (0.18–0.59 g/day)	None	121	6.23 (5.82, 6.63)	Reference
	Any	25	6.16 (5.50, 6.83)	−0.07 (−1.31, 0.62); *p* = 0.51
p for interaction: 0.48 ^d^				
**Vitamin C**				
High (44.60–1250.35 mg/day)	None	374	6.11 (5.90, 6.32)	Reference
	Any	64	6.19 (5.75, 6.64)	0.07 (−0.45, 0.59); *p* = 0.79
Low (5.58–44.59 mg/day)	None	105	6.04 (5.67, 6.41)	Reference
	Any	31	6.65 (5.89, 7.41)	0.67 (−0.16, 1.51); *p* = 0.11
*p* for interaction: 0.28				
**Vitamin E**				
High (4.07–415.69 mg/day)	None	361	6.08 (5.87, 6.29)	Reference
	Any	64	6.35 (5.83, 6.87)	0.31 (−0.29, 0.91); *p* = 0.31
Low (0.80–4.06 mg/day)	None	117	6.26 (5.83, 6.57)	Reference
	Any	31	6.15 (5.57, 6.74)	−0.10 (−0.90, 0.55); *p* = 0.64
*p* for interaction: 0.48				
**Fiber**				
High (8.70–42.33 g/day)	None	368	6.14 (5.93, 6.36)	Reference
	Any	58	6.32 (5.81, 6.84)	0.19 (−0.41, 0.79); *p* = 0.53
Low (2.58–8.69 g/day)	None	111	5.94 (5.64, 6.24)	Reference
	Any	37	6.20 (5.59, 6.81)	0.28 (−0.42, 0.98); *p* = 0.44
*p* for interaction: 0.86				

Abbreviations: CI, confidence interval; HbA1c, glycated hemoglobin. ^a^ The linear regression models adjusted for sex, dialect, date of birth, date of recruitment and date of biospecimen collection. ^b^ Nutrient categories were low (lowest quartile) and high (second through fourth quartiles). ^c^ In addition to the minimal set of covariates, the fully adjusted model included education and BMI at follow-up in the model. ^d^ The *p*-values for interaction were generated by adding a product term between self-report of household smokers and nutrient categories into separate models.
